# Penetrating aortic injury left untreated for 20 days: a case report

**DOI:** 10.1186/s12893-018-0337-z

**Published:** 2018-01-27

**Authors:** Alessia Giaquinta, Dovile Mociskyte, Giuseppe D’Arrigo, Giuseppe Barbagallo, Francesco Certo, Massimiliano Veroux, Pierfrancesco Veroux

**Affiliations:** 1 Vascular Surgery and Organ Transplant Unit, Department of Surgery, University Hospital of Catania, Via Santa Sofia, 84 95123 Catania, Italy; 2 Neurosurgery Unit, Department of Surgery, University Hospital of Catania, Catania, Italy

**Keywords:** Penetrating trauma, Aorta, Bullet, Late diagnosis, Gunshot injury, Firearm lesion, Aortic injury

## Abstract

**Background:**

Penetrating aortic trauma remains one of the most challenging injuries with a high mortality rate if left untreated, or if the surgical treatment is delayed. We present an uncommon case of a late diagnosed abdominal firearm injury, in which the bullet partially penetrated the wall of the aorta, creating a plug that prevented immediate death due to massive bleeding.

**Case presentation:**

A 26-year-old Libyan man was a victim of a firearm wound, with a bullet penetrating his abdominal wall from the left to right side. After the assault, the victim, spent up to 20 days crossing the Mediterranean Sea to leave his country of origin. Abdominal radiography revealed the presence of a bullet located anteriorly to the second lumbar vertebra, while computed tomography angiography, unexpectedly, demonstrated that the bullet penetrated partially into the aortic wall at the level of the left renal artery. The bullet penetrated the aortic wall for half of its length, creating a plug that avoided immediate life-threatening bleeding at the time of the gunshot injury. The bullet was removed and the aortic lesion was repaired. The patient was discharged 6 days after the surgical procedure, in good health.

**Conclusions:**

We presented a very rare case of late diagnosis of aortic injury caused by a gunshot lesion, in which the particular trajectory of the bullet helped avoid immediate life-threatening bleeding and, probably, saved the patient’s life.

**Electronic supplementary material:**

The online version of this article (10.1186/s12893-018-0337-z) contains supplementary material, which is available to authorized users.

## Background

Penetrating aortic trauma remains one of the most challenging injuries; the mortality rate is as high as 87.5% for gunshot lesions [1], with most patients dying upon admittance to the emergency room due to uncontrolled bleeding. There are many possible clinical scenarios following a gunshot wound evaluation, such as aortocaval fistula, aortic pseudoaneurysm formation, or peripheral bullet embolization [2]. In almost all cases, prompt surgical treatment is required to prevent an immediate death. Here, we present a very rare case involving a patient with a firearm injury to the abdomen, treated 20 days after the assault, with the bullet partially penetrating the wall of the aorta and creating a plug that prevented immediate death due to massive bleeding.

## Case presentation

A 26-year-old Libyan man was a victim of a firearm wound while he was running seeking for refuge, with a bullet penetrating his abdominal wall from the left to right side, with an entrance orifice of approximately 3 cm in diameter at the left lumbar paravertebral region. No exit wound was seen. After the assault, the victim, a clandestine refugee from Libya, spent up to 20 days crossing the Mediterranean Sea to leave his country of origin. He was finally admitted to a recovery center in Italy and then to a local hospital, in good general condition and with no signs of hemodynamic shock.

**Fig. 1:**
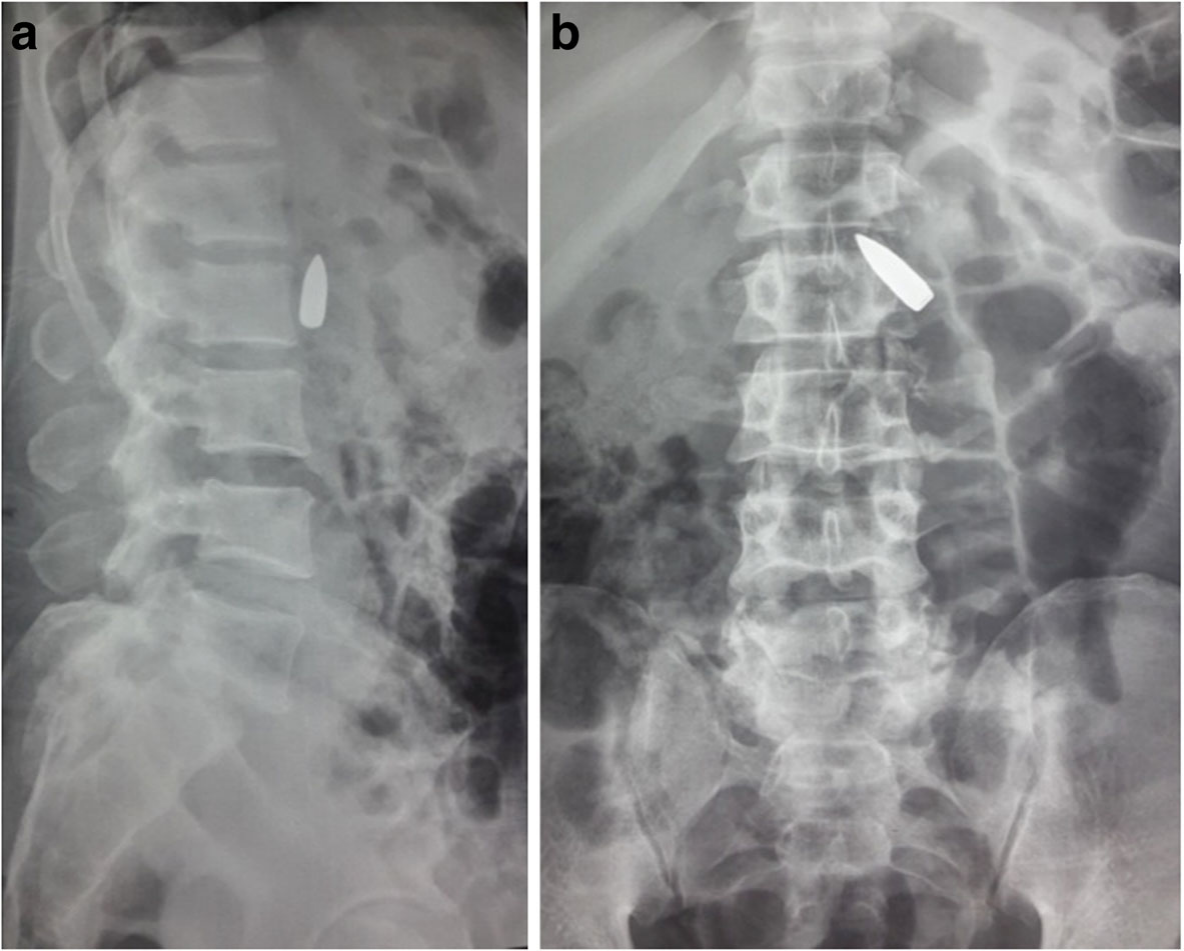
Abdominal radiography in the lateral (**a**) and front (**b**) positions demonstrated the presence of a bullet located anteriorly to the second lumbar vertebra, with the tip rotated in the upright direction

**Fig. 2:**
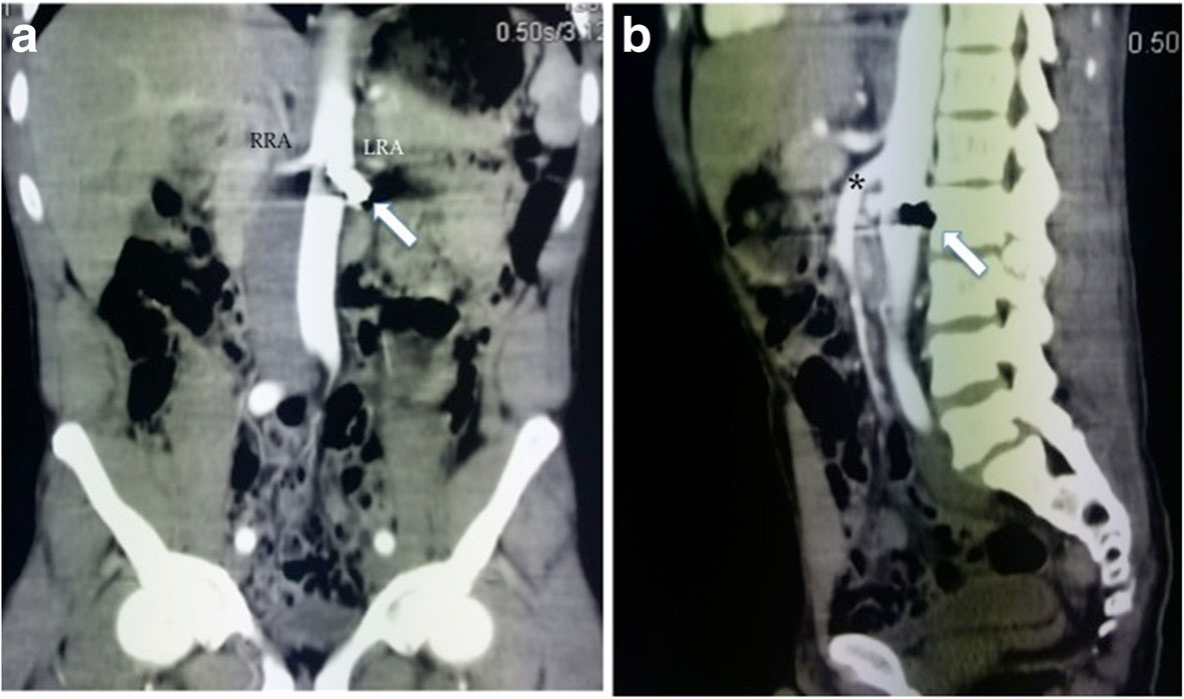
Abdominal computed tomography angiography demonstrated the presence of a bullet (arrow) partially penetrating the aortic wall at the level of the right renal artery (RRA) (**a**) close to the origin of the left renal artery (LRA), just below the superior mesenteric artery (*) (**b**)

**Fig. 3:**
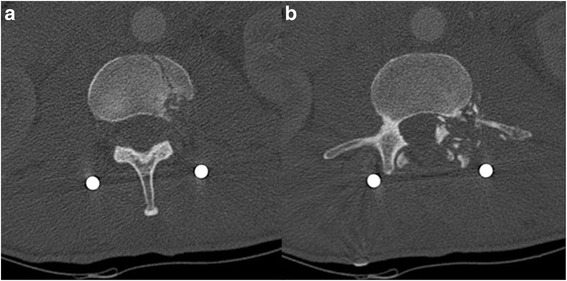
Abdominal computed tomography axial view demonstrating the significant injury to the body (**a**) and to the pedicle (**b**) and posterior arch of the lumbar vertebrae, supporting the opinion given that the vertebra dissipated the high velocity kinetic energy of the bullet

Abdominal radiography revealed the presence of a bullet located anteriorly to the second lumbar vertebra, with the tip rotated in the upright direction and fractures of the second and third lumbar vertebrae (Fig. 1). Subsequently, the patient was immediately transferred to the department of neurosurgery of our hospital. At admission, he was hemodynamically stable, with a stable hemoglobin value (10.1 g/dl), a Glasgow Coma Scale of 15, and with lumbar pain, hyposthenia, and hyperesthesia of the left lower limb. Preoperative computed tomography (CT) angiography, unexpectedly, demonstrated that the bullet penetrated partially into the aortic wall at the level of the left renal artery, without any signs of active blood loss or surrounding hematoma (Figs. 2, 3).

**Fig. 4:**
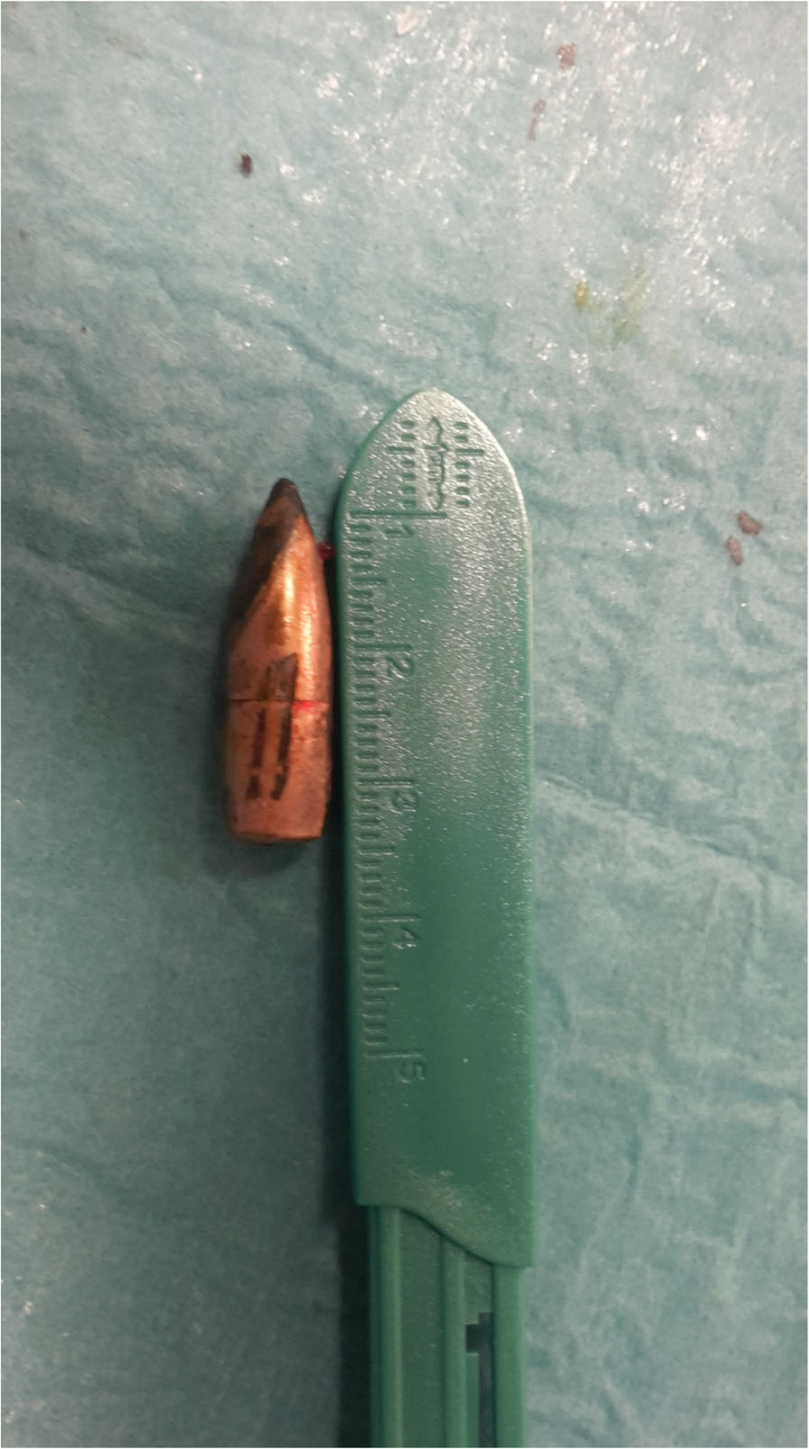
The bullet after its extraction from the aortic wall

The patient was immediately scheduled for surgical repair of the aortic injury. The aorta was accessed retroperitoneally through a left lobotomy. The abdominal aortic segment was entirely exposed by a retrorenal access and section of the fibers of the left pillar of the diaphragm and then isolated before dealing with the bullet. At the level of the left renal artery, a bullet partially penetrating the left latero-posterior aortic wall was visualized. The bullet crossed the lumbar spine, fragmenting the second and third lumbar vertebrae, and finally penetrated the aortic wall for half of its length, creating a plug that avoided immediate life-threatening bleeding at the time of the gunshot injury. There were no signs of visceral injury or surrounding hematoma. After clamping of the aorta and the left renal artery, the bullet was clearly visible on the posterior surface of the aorta, just behind the left renal artery (Video 1). Subsequently, it was removed and the aortic lesion was repaired by a 5–0 polypropylene running suture. The extracted bullet was about 27 mm in length, with a spritzer (pointed) nose, resembling a military rifle cartridge (Fig. 4). The surgical intervention was completed by the neurosurgery team by using spinal fixation with pedicle screws at the level between the second and fourth lumbar vertebrae. The immediate postoperative course was uneventful, and computed tomography angiography performed on postoperative day 3 did not demonstrate any sign of aortic bleeding and showed good results of the spinal fixation. Consequently, the patient was discharged 6 days after the surgical procedure, without any neurological symptoms.Additional file 1:Video 1. After clamping, the aorta was dissected and the bullet was clearly visible on the posterior surface of the aortic wall. (M4V 2928 kb)

## Discussion and conclusion

The anatomic site, organs involved, and the ballistic properties of the penetrating projectiles determine the severity of the lesion after an aortic injury. If the bullet is small enough, the elasticity of the wall of the aorta may result in a spontaneous and immediate closure of the entry wound. The bullet may also embolize to distal arteries, thereby leading to critical ischemia. The incidence of this is, however, very low, with fewer than 200 cases reported [3, 4]. CT scan is the preferred method to investigate the layers of the aortic wall, although in selected case angiography and/or intravascular ultrasound could be used to eliminate scatter from the bullet for more sensitive diagnosis.

The case described herein is very uncommon, because the unusual trajectory of the bullet through the lumbar spine and finally the aortic wall resulted in a delayed diagnosis, with potentially life-threatening-complications.

The extracted bullet resembled a military rifle cartridge. Most modern military (assault) rifles launch their projectiles at 700–960 m/s and are defined as “high velocity”. The effects of rifle bullets can be far more destructive compared to handguns because of their higher energy [5–8] and tendency to change their orientation (by becoming unstable and “yawing” or turning sideways relative to the line of flight) [5]. Another important wounding mechanism besides direct tissue damage is dynamic temporary cavity formation, which becomes clinically important at impact velocities exceeding 600 m/s [9]; this mechanism is thus considered one of the most important features in wound ballistics of high-velocity projectiles [5].

Considering these characteristics, in the present patient, the bone injury was probably responsible for the retardation of the penetrating bullet [7], resulting in a loss of the kinetic energy and enabling the bullet to pierce the abdominal aorta and get stuck between the muscular and elastic layers of the aortic wall. The pointed shape of the nose and steel jacket of the bullet are physical properties that may also be associated with minor tissue damage, as the bullet does not deform in the tissue and has a longer distance before beginning to jaw [6]. The high temperature of the penetrating bullet, resulting in cauterization of the adjacent tissues, may also have prevented hematoma formation and infection. Thus, despite the complicated bullet position and quite complex surgical treatment, the final result was optimal, without any postoperative complications or neurological sequelae.

In conclusion, we presented a very rare case of late diagnosis of aortic injury caused by a gunshot lesion, in which the particular trajectory of the bullet helped avoid immediate life-threatening bleeding and, probably, saved the patient’s life.
